# Analysis of Course of Changes in Blood Lactate Concentration in Response to Graded Exercise Test and Modified Wingate Test in Adolescent Road Cyclists

**DOI:** 10.3390/jcm13020535

**Published:** 2024-01-17

**Authors:** Bartosz Zając

**Affiliations:** Laboratory of Functional Diagnostics, Central Scientific and Research Laboratory, University of Physical Education in Kraków, 31-571 Kraków, Poland; bartosz.zajac@awf.krakow.pl; Tel.: +48-508-116-286

**Keywords:** blood lactate concentration, graded exercise test, modified Wingate test, road cyclist

## Abstract

Background: The purpose of this study was to analyze the course of changes in the blood lactate (BL) concentration in response to the graded exercise test (GXT) and the modified Wingate test (MWT). Methods: This study involved 23 male highly trained road cyclists (age: 16.2 ± 1.1 years; experience: 5.0 ± 2.1 years; VO_2_max 59.0 ± 3.5 mL × kg^−1^ × min^−1^). The analysis of BL concentration was conducted using an enzymatic–amperometric electrochemical technique. Results: Our study provided the following information: (i) peak BL concentration in response to GXT (12.86 ± 2.32 mmol × L^−1^) and MWT (12.85 ± 1.47 mmol × L^−1^) is expected around the third minute after the completion of the trial; (ii) 60 min is not a sufficient period for BL concentration to return to resting values after GXT; (iii) post-GXT BL removal during the 60 min period is unsteady (3–20 min: −2.6 ± −0.6% × min^−1^; 20–60 min: −1.6 ± −0.3% × min^−1^; *p*-value for comparison < 0.01), whereas post-MWT BL removal during the 12 min period appears to be constant (3–6 min: −2.4 ± −5.6% × min^−1^, 6–9 min: −2.6 ± −1.8 % × min^−1^; 9–12 min: −3.1 ± −2.1 % × min^−1^; *p*-value for all comparisons < 0.01). Conclusions: When aiming to obtain valuable data regarding the course of changes in BL concentration during the post-exertion period, it is essential to consider the number of measurements and the time points in sample collection for analysis.

## 1. Introduction

The direct source of energy for muscle work is the breakdown of adenosine triphosphate (ATP). The ATP content in a muscle cell (~5 mmol × kg^−1^ of wet muscle) is sufficient for only a few maximal muscle contractions. The depletion of ATP in muscle cells leads to the permanent binding of actin and myosin filaments, a state that occurs after the cessation of life processes, resulting in post-mortem rigor mortis. In a living organism, even during very intense exertion, the ATP reserves in muscle cells are only depleted by ~20% of the initial content. This is because the increases in adenosine diphosphate (ADP) and inorganic phosphate (P), as well as the decrease in ATP content, activate a series of biochemical processes, leading to ATP resynthesis. Some of these processes occur in the cytoplasm of muscle cells and do not require oxygen (anaerobic processes), while others take place in mitochondria with the involvement of oxygen (aerobic processes). Resynthesis involves the attachment of P to ADP. This process requires energy derived from the breakdown of chemical bonds in compounds, such as phosphocreatine, carbohydrates, fats, and proteins [1].

Thanks to the presence of anaerobic processes, muscles can work before the functions involved in oxygen transport reach a level that meets the demand, and they can overcome loads where the demand for oxygen exceeds the body’s oxygen uptake capacity. Among the most important anaerobic processes occurring in working muscles are the hydrolysis of phosphocreatine and glycolysis. The content of phosphocreatine in muscles at rest is about 26 mmol × kg^−1^ of wet muscle [2]. During very intense efforts, such as sprinting, this reserve is completely depleted. The continuation of work is possible due to anaerobic glycolysis. In this process, the glycogen stored in muscle cells is utilized for the resynthesis of ATP. Glycogen reserves are substantial (approximately 80–120 mmol × kg^−1^ of wet muscle) [3]; however, a muscle can only function under anaerobic conditions for 0.5–2 min because working in these conditions involves a rapid accumulation of H^+^ ions, leading to a decrease in pH [4]. The acidification of the cellular environment contributes to the inhibition of the rate of ATP hydrolysis due to an impaired contractile apparatus function and electromechanical coupling [5]. Lactate produced during glycolysis and H^+^ ions diffuses from cells into the blood. During intense exertion, the production rate is higher than the diffusion rate, causing these products to accumulate in the muscles. After the end of work or a reduction in intensity, lactate is oxidized by the liver, where it undergoes transformation into glucose. This transformation occurs within the Cori cycle.

The measurement of the blood lactate (BL) concentration is widely used for assessing the involvement of anaerobic glycolysis in providing energy for muscle work and, indirectly, for evaluating exercise intensity. Measurements of BL concentration are commonly utilized in standardized procedures, such as exercise tests. They serve to determine metabolic thresholds [6,7] and act as criteria for reaching maximal oxygen uptake (VO_2_max) in graded exercise tests (GXTs) [8,9]. They also function as indicators of the potential activation of anaerobic glycolysis during short-term, high-intensity all-out efforts [10,11,12]. In training practice, BL concentration measurements are used to monitor the intensity of exercise [13,14] and the post-exertional recovery status [15,16,17].

According to the author, significantly less attention (in relation to the applications mentioned above) has been given in the literature to the course of changes in BL concentration during the post-exertional period [11,18,19,20]. Particularly, little is known about the course of these changes in the population of highly trained adolescent road cyclists, whose bodies often have to cope with the physiological effects of aerobic and anaerobic muscle work during training [21,22] and sports competitions [23,24]. What is known, however, is that the course of changes in BL concentration in response to exercise depends on the sports specialization [25], the level of biological development [26], and that it is modifiable through sports training [27,28], being associated with sports performance [29,30]. Therefore, acquiring such information can be valuable from the perspective of sports science and training practice. For example, these data can help understand the expected values of BL concentration in this population, as well as when, how much, and at what moment measurements should be taken to achieve the desired parameters. Taking the above into consideration, the purpose of this study was to analyze the course of changes in BL concentration in response to the GXT and MWT in highly trained adolescent road cyclists.

## 2. Materials and Methods

### 2.1. Study Design

**Figure 1:**
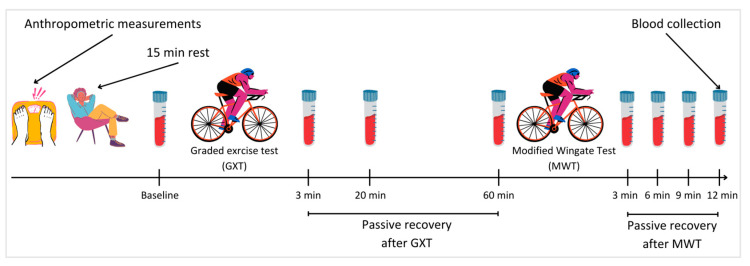
Course of the study.

The examination plan included conducting GXT, MWT, anthropometric measurements, and 8 BL concentration measurements for each participant. The detailed protocol is outlined in Figure 1. One iteration of the entire process was carried out for each athlete. All activities were conducted between 10:00 a.m. and 4:00 p.m. under ambient conditions, with a temperature of 20 ± 1 °C and a relative humidity of 40 ± 5%. Before each test, cyclists familiarized themselves with the testing procedures, and their execution was supervised by the researcher. GXT and MWT were carried out using a Cyclus 2 ergometer (RBM electronic-automation GmbH, Leipzig, Germany) with the athlete’s own bike installed—the same bike used for training and competing in races. The ergometer was calibrated following the manufacturer’s recommendations. Participants were instructed not to consume stimulants (e.g., caffeine) on the test day, to avoid heavy training sessions 36–48 h before testing, and to refrain from altering their dietary habits before the study. All procedures adhered to the principles outlined in the 1964 Declaration of Helsinki and its subsequent amendments. Consent for testing was obtained from the Bioethics Committee at the Regional Medical Chamber in Kraków (No. 249/KBL/OIL/2021, 17 September 2021).

### 2.2. Participants

This study involved 23 male road cyclists (age: 16.2 ± 1.1 years; body height 177.4 ± 5.8 cm; body mass 64.5 ± 6.3 kg; body fat 15.9 ± 2.2%; training experience: 5.0 ± 2.1 years) recruited from students of the Sports Championship School of Cycling. All students meeting the eligibility criteria participated in the study. The inclusion criteria were as follows: (i) progression of biological development—minimum 3rd pubic hair stage on Tanner’s Scale [31]; (ii) “highly trained/national elite” level according to McKay’s participant classification framework for research in sports sciences [32]; (iii) having a current certificate from a sports medicine doctor regarding the ability to practice road cycling. The exclusion criteria included age above 18 years or a health condition that could impair performance, such as illness, injury, or mental health issues. The participants and their legal guardians were provided with detailed information about the research protocol and subsequently provided written informed consent to participate in the study.

### 2.3. Anthropometric Measurements

Body height was measured using a stadiometer (seca 213, seca gmbh & co. kg, Hamburg, Germany) with an accuracy of 1 mm. Body mass, fat mass, and lean body mass were assessed using a body composition multi-frequency (5 kHz/50 kHz/250 kHz) octopolar analyzer (MC 780 MA, Tanita, Tokyo, Japan) based on the electrical bioimpedance method. The measurements were performed following the conditions recommended by the manufacturer of the analyzer.

### 2.4. Graded Exercise Test

The GXT began with 4 min warm-up performed at 80 W. Next, the load was increased by 40 W every 2 min. The effort was continued until volitional exhaustion, which was manifested in the inability to maintain a cadence higher than 70 rpm. After completing the test, participants engaged in a 2 min exercise at an intensity of 50 W. During testing, the following cardiopulmonary variables were measured breath by breath via the Quark CPET ergospirometer (Cosmed, Rome, Italy): minute ventilation (VE), oxygen uptake (VO_2_) and carbon dioxide production (VCO_2_), heart rate (HR). Recorded data were averaged across 10 s intervals. The ergospirometer was calibrated according to the manufacturer’s instructions. Rating of perceived exertion (RPE) was assessed in the last 15 s of each 2 min interval using 6–20 on the Borg scale [33]. Based on the measured variables, the maximum minute oxygen consumption (VO_2_max) was determined. The following criteria were used for VO_2_max determination: an increase in VO_2_ of <150 mL × min^−1^ with an increase in power, respiratory exchange ratio > 1.10, RPE > 18 on Borg’s Scale, HR > 90% age-predicted maximal HR calculated according to Tanaka [8,34].

### 2.5. Modified Wingate Test

At baseline, participants performed a 4 min warm-up at 90 W [35]. During the final 3–5 s of the second and fourth minutes of the warm-up, athletes executed maximal accelerations. Two minutes after the warm-up, participants engaged in a maximal 30 s all-out effort with a stationary start. Following the completion of the test, participants performed a 2 min exercise at an intensity of 50 W. The resistance applied during the test was set at 10% body weight [36]. Upon the “Go!” command, participants were instructed to achieve the maximum pedaling rate as quickly as possible and then maintain it in a seated position until the end of the effort, with strong verbal encouragement. Anaerobic performance indicators, including peak power, time to obtain peak power, mean power, and fatigue index, were determined based on measurements recorded by the ergometer [35].

### 2.6. Blood Lactate Concentration Measurement

To measure BL concentration, the Super GL2 analyzer manufactured by Müller Gerätebau GmbH in Freital, Germany, was utilized. The analysis employed an enzymatic–amperometric electrochemical technique. The lactate analyzer was calibrated before each analysis session for every participant according to the manufacturer’s recommendations. For the measurements, 20 µL blood samples were drawn into a capillary from the fingertip and punctured using disposable lancets. BL removal index (BLRI) was calculated according to the formula:

$$BLRI[\%\times {min}^{-1}]=\left(\frac{B-A}{A}\times 100\%\right)÷∆T$$

A—BL concentration [mmol × L^−1^] at time point A;
B—BL concentration [mmol × L^−1^] at the time point B;
∆T—time interval in minutes between measurements A and B;
Assumption: point A precedes point B.

### 2.7. Statistical Analysis

The statistical analysis was conducted using Statistica 13.3 software (TIBCO Software Inc., Palo Alto, CA, USA). ANOVA with repeated measures was employed to assess differences in lactate concentration across successive measurements. Prior to conducting the analysis, assumptions of normality of distributions (Shapiro–Wilk test), homogeneity of variances (Levene’s test), and sphericity (Mauchly’s test) were verified. In cases where the assumption of sphericity was violated, Greenhouse–Geisser correction was applied. For post hoc analysis, the Tukey’s (HSD) test was employed. The statistical significance level was set at two levels, namely 0.05 and 0.01, representing the probabilities of Type I error.

## 3. Results

### 3.1. Aerobic and Anaerobic Performance

Indicators of aerobic and anaerobic performance obtained during GXT and MWT are presented in Table 1 and Table 2, respectively.

### 3.2. Post-GXT Course of Changes in BL Concentration

**Figure 2:**
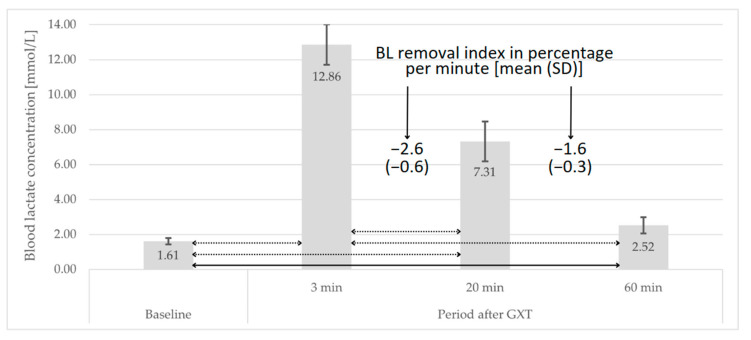
BL concentration under baseline and post-GXT period; bar—mean, whiskers—standard deviation, solid line—*p*-value < 0.05, dotted line—*p*-value < 0.01.

The peak BL concentration in the post-GXT period was observed in the 3rd minute. Sixty minutes was insufficient to restore the BL concentration to the initial values. A statistically significantly (*p*-value < 0.01) higher BL removal rate was demonstrated between 3 and 20 min compared to the BL removal rate between 20 and 60 min. Detailed data on the course of BL concentration values are presented in Figure 2.

### 3.3. Post-MWT Course of Changes in BL Concentration

**Figure 3:**
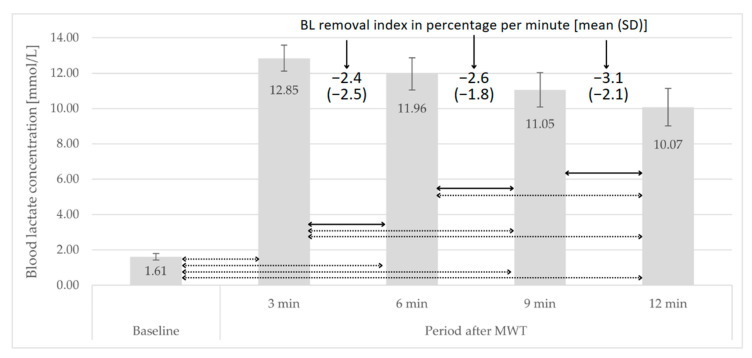
BL concentration under baseline and post-MWT period; bar—mean, whiskers—standard deviation, solid line—*p*-value < 0.05, dotted line—*p*-value < 0.01.

The peak BL concentration in the post-MWT period was observed in the 3rd minute. No statistically significant differences were found (*p*-values for all comparisons > 0.05) in the BL removal rate across successive time intervals. Detailed data on course of BL concentration values are presented in Figure 3.

## 4. Discussion

The purpose of this study was to analyze the course of changes in BL concentration in response to the GXT and the MWT. According to the author’s best knowledge, this is one of the first studies of its kind conducted among a group of high-trained male adolescent road cyclists. Taking into account the adopted schedule of measurements, this study provided the following information: (i) peak BL concentration in response to GXT and MWT is expected around the 3rd minute after the completion of the trial; (ii) 60 min is not a sufficient period for BL concentration to return to resting values after GXT; (iii) the BL removal rate after GXT during the 60 min post-test period is unsteady, whereas the BL removal rate after MWT during the 12 min post-test period appears to be constant.

The peak BL concentration in response to GXT and MWT, according to the methodology adopted by the author, occurred in 3 min and averaged approximately 12.9 mmol × L^−1^ in both cases. The results of Beneke et al.’s [26] study indicate that a significant factor influencing the magnitude of changes in BL concentration in response to GXT and the Wingate test is the level of biological development. These authors observed that children, in comparison to adolescents and adults, exhibit significantly lower post-exercise peak values of BL concentration. In a study by Beneke et al. [26], the mean peak BL concentration in adolescents after GXT was 12.0 mmol × L^−1^, and after the Wingate test, it was 12.7 mmol × L^−1^. Similar observations were obtained by other researchers investigating this issue [37,38]. In light of the above, there is no basis for rejecting the hypothesis put forward by some researchers that the rate of muscular glycolysis increases with maturation [39,40]. On the other hand, Mero [41], investigating pre-pubertal trained boys (engaged in weightlifting, tennis, endurance running, and sprinting), recorded an average peak BL concentration of 13.1 mmol × L^−1^ in the 5th minute of recovery after an all-out 60 s trial and 7.9 mmol × L^−1^ after GXT. Higher BL concentration values (despite the earlier developmental stage of subjects) after the all-out trial and lower values after the GXT obtained by Mero, in comparison to the results of our own studies, may be attributed to the heterogeneity of pre-pubertal boys in terms of sports specialization. In other words, the study participants in Mero’s research were boys representing endurance and strength–speed disciplines. Achieving high results in a specific sports specialization depends largely on physiological predispositions as well as commitment to a systematic and targeted training process [42]. One of such physiological factors is the composition of muscle fiber types, which is associated with the production and utilization of lactate [41]. Consequently, in endurance sports, high athletic performance will be achieved by individuals with a high percentage of slow-twitch I (slow-oxidative) muscle fibers, while in speed–strength sports, individuals with a high percentage of fast-twitch IIA (fast-oxidative glycolytic) and fast-twitch IIX (fast glycolytic) fibers will excel [43,44,45,46].

In this study, the post-GXT BL removal rate between 3 and 20 min averaged 2.6% × min^−1^ and was significantly higher than the rate between 20 and 60 min, which reached an average value of 1.6% × min^−1^. On the other hand, the BL removal rate after MWT between 3 and 12 min was relatively constant, reaching an average value of 3% × min^−1^. The results in this area indicate that the course of post-exercise changes in BL concentration, as well as peak values, depends on biological development and the composition of muscle fibers. Interestingly, Beneke et al. [26] observed that the rate of BL removal in boys, compared to adolescents and adults, is 30% higher. The comparison of our own research results with those obtained by Mero [41] (average BL removal rate between 5 and 15 min approx. −6.6% × min^−1^ after an all-out 60-s trial) is consistent in terms of the direction of differences observed by Beneke’s team. Moreover, other researchers have observed that the course of changes in BL concentration during the post-exercise period is modifiable through sports training [27,28] and is also associated with sports performance [29]. When it comes to the course of changes in the BL removal rate, the results of our own research align with the reports of other authors, indicating that the course of changes in this variable is very well fitted to a bi-exponential model (r^2^ > 0.99) [19,47].

The information obtained in this study may have certain practical applications. For example, to register the peak BL concentration in response to MWT (e.g., as a diagnostic criterion) or GXT (e.g., as one of the criteria for achieving VO_2_max), measurements should be taken around the 3rd minute after the completion of the test. The study’s author speculates that this principle may be applicable in training practice, especially for training carried out at an intensity corresponding to VO_2_max or higher. The study results seem to have another valuable practical application from both scientific and practitioner perspectives. Namely, both groups may be interested in the BL removal rate index for various reasons. Assuming that this rate remains relatively constant between the 3rd and 12th minutes after exercise, one can easily obtain such an index based on two blood samples from the specified period and knowledge of the amount of time that separated their collection.

The main limitation of this study was the failure to measure BL concentration immediately after the completion of the trials, leaving uncertainty regarding the magnitude and direction of changes in the first 3 min post-test. Another limitation is the lack of BL concentration measurements after 12 min from the completion MWT. Perhaps additional measurements would have allowed us to determine the point at which the BL removal rate begins to decrease, as observed in the post-GXT period. One limitation of this study may be the use of passive rest following exertion. Adopting such a methodology is likely to result in lower BL removal rates compared to the situation where active rest would have been applied [48,49]. Additionally, this approach may lead to a less precise representation of sports competition conditions, during which cyclists must continue their efforts despite the effects of fatigue. Moreover, among the limitations of this study, one can mention the homogeneous, in terms of gender, study group; therefore, the results cannot be generalized to the female population.

## 5. Conclusions

From the perspective of aiming to obtain valuable data regarding the course of changes in BL concentration during the post-exertion period, it is essential to consider the number of measurements and the time points for sample collection for analysis. In future studies in this field, it is advisable to consider conducting measurements with higher sampling frequency (e.g., every 1–2 min) and/or over a more extended post-exertion period (exceeding 60 min).

## Figures and Tables

**Table 1:** Indicators of aerobic performance obtained in the GXT.

	Mean ± SD	CV (%)	Minimum–Maximum
P_VO2MAX_ (W)	379 ± 33	8.7	320–440
P_VO2MAX_ (W × kg^−1^)	5.90 ± 0.44	7.5	4.87–6.90
VO_2_max (L × min^−1^)	3.79 ± 0.29	7.7	3.22–4.51
VO_2_max (mL × kg^−1^ × min^−1^)	59.0 ± 3.5	5.9	48.5–65.9

SD—standard deviation, CV—coefficient of variation, P_VO2MAX_—power at VO_2_max level.

**Table 2:** Indicators of anerobic performance obtained in the MWT.

	Mean ± SD	CV (%)	Minimum–Maximum
Peak power (W)	923 ± 111	12.0	710–1232
Peak power (W × kg^−1^)	14.3 ± 0.6	4.2	13.4–15.6
Time to obtain peak power (s)	3.16 ± 0.99	31.3	1.00–5.10
Mean power (W)	722 ± 81	11.2	553–894
Mean power (W × kg^−1^)	11.2 ± 0.6	5.4	10.3–12.6
Fatigue index (%)	42.9 ± 6.7	15.6	31.1–56.3

SD—standard deviation, CV—coefficient of variation.

## Data Availability

The data presented in this study can be obtained by contacting the corresponding author upon request.

## References

[1] Hargreaves M., Spriet L.L. (2020). Skeletal Muscle Energy Metabolism during Exercise. Nat. Metab..

[2] Baker J.S., McCormick M.C., Robergs R.A. (2010). Interaction among Skeletal Muscle Metabolic Energy Systems during Intense Exercise. J. Nutr. Metab..

[3] Bergström J., Hultman E., Roch-Norlund A.E. (1972). Muscle Glycogen Synthetase in Normal Subjects. Scand. J. Clin. Lab. Investig..

[4] Cheetham M.E., Boobis L.H., Brooks S., Williams C. (1986). Human Muscle Metabolism during Sprint Running. J. Appl. Physiol..

[5] Westerblad H., Lee J.A., Lannergren J., Allen D.G. (1991). Cellular Mechanisms of Fatigue in Skeletal Muscle. Am. J. Physiol.-Cell Physiol..

[6] Binder R.K., Wonisch M., Corra U., Cohen-Solal A., Vanhees L., Saner H., Schmid J.-P. (2008). Methodological Approach to the First and Second Lactate Threshold in Incremental Cardiopulmonary Exercise Testing. Eur. J. Cardiovasc. Prev. Rehabil..

[7] Pallarés J.G., Morán-Navarro R., Ortega J.F., Fernández-Elías V.E., Mora-Rodriguez R. (2016). Validity and Reliability of Ventilatory and Blood Lactate Thresholds in Well-Trained Cyclists. PLoS ONE.

[8] Midgley A.W., McNaughton L.R., Polman R., Marchant D. (2007). Criteria for Determination of Maximal Oxygen Uptake. Sports Med..

[9] Edvardsen E., Hem E., Anderssen S.A. (2014). End Criteria for Reaching Maximal Oxygen Uptake Must Be Strict and Adjusted to Sex and Age: A Cross-Sectional Study. PLoS ONE.

[10] Beneke R., Pollmann C., Bleif I., Leithäuser R., Hütler M. (2002). How Anaerobic Is the Wingate Anaerobic Test for Humans?. Eur. J. Appl. Physiol..

[11] Takei N., Kakinoki K., Hatta H. (2020). Repeated Sprint Training in Hypoxia Delays Fatigue during 30-Sec All-out Sprint and Reduces Blood Lactate Concentrations after Exercise in Trained Cyclists: A Case Study. J. Phys. Fit. Sports Med..

[12] Paixão R., da Mota G., Marocolo M. (2014). Acute Effect of Ischemic Preconditioning Is Detrimental to Anaerobic Performance in Cyclists. Int. J. Sports Med..

[13] Beneke R., Leithäuser R.M., Ochentel O. (2011). Blood Lactate Diagnostics in Exercise Testing and Training. Int. J. Sports Physiol. Perform..

[14] Granata C., Jamnick N.A., Bishop D.J. (2018). Principles of Exercise Prescription, and How They Influence Exercise-Induced Changes of Transcription Factors and Other Regulators of Mitochondrial Biogenesis. Sports Med..

[15] Franchini E., de Moraes Bertuzzi R.C., Takito M.Y., Kiss M.A.P.D.M. (2009). Effects of Recovery Type after a Judo Match on Blood Lactate and Performance in Specific and Non-Specific Judo Tasks. Eur. J. Appl. Physiol..

[16] Martin J.S., Friedenreich Z.D., Borges A.R., Roberts M.D. (2015). Acute Effects of Peristaltic Pneumatic Compression on Repeated Anaerobic Exercise Performance and Blood Lactate Clearance. J. Strength Cond. Res..

[17] Di Masi F., De Souza Vale R.G., Dantas E.H.M., Barreto A.C.L., da Silva Novaes J., Reis V.M. (2007). Is Blood Lactate Removal during Water Immersed Cycling Faster than during Cycling on Land?. J. Sports Sci. Med..

[18] Ferreira J., Da Silva Carvalho R., Barroso T., Szmuchrowski L., Śledziewski D. (2011). Effect of Different Types of Recovery on Blood Lactate Removal After Maximum Exercise. PJST.

[19] Durand R., Galli M., Chenavard M., Bandiera D., Freund H., Messonnier L.A. (2021). Modelling of Blood Lactate Time-Courses During Exercise and/or the Subsequent Recovery: Limitations and Few Perspectives. Front. Physiol..

[20] Messonnier L.A., Emhoff C.-A.W., Fattor J.A., Horning M.A., Carlson T.J., Brooks G.A. (2013). Lactate Kinetics at the Lactate Threshold in Trained and Untrained Men. J. Appl. Physiol..

[21] Zając B., Gaj P., Ambroży T. (2023). Analysis of Training Loads in Polish Adolescent Road Cyclists in the Preparatory Period and Their Effects on Physical Fitness. J. Kinesiol. Exerc. Sci..

[22] Faria E.W., Parker D.L., Faria I.E. (2005). The Science of Cycling. Sports Med..

[23] Ebert T.R., Martin D.T., Stephens B., Withers R.T. (2006). Power Output During a Professional Men’s Road-Cycling Tour. Int. J. Sports Physiol. Perform..

[24] Gallo G., Leo P., March M.M., Giorgi A., Faelli E., Ruggeri P., Mujika I., Filipas L. (2022). Differences in Training Characteristics Between Junior, Under 23 and Professional Cyclists. Int. J. Sports Med..

[25] Bret C., Messonnier L., Nouck Nouck J.M., Freund H., Dufour A.B., Lacour J.R. (2003). Differences in Lactate Exchange and Removal Abilities in Athletes Specialised in Different Track Running Events (100 to 1500 m). Int. J. Sports Med..

[26] Beneke R., Hütler M., Jung M., Leithäuser R.M. (2005). Modeling the Blood Lactate Kinetics at Maximal Short-Term Exercise Conditions in Children, Adolescents, and Adults. J. Appl. Physiol..

[27] Messonnier L., Freund H., Féasson L., Prieur F., Castells J., Denis C., Linossier M.-T., Geyssant A., Lacour J.-R. (2001). Blood Lactate Exchange and Removal Abilities after Relative High-Intensity Exercise: Effects of Training in Normoxia and Hypoxia. Eur. J. Appl. Physiol..

[28] Messonnier L., Freund H., Denis C., Féasson L., Lacour J.-R. (2006). Effects of Training on Lactate Kinetics Parameters and Their Influence on Short High-Intensity Exercise Performance. Int. J. Sports Med..

[29] Messonnier L., Freund H., Bourdin M., Belli A., Lacour J.-R. (1997). Lactate Exchange and Removal Abilities in Rowing Performance. Med. Sci. Sports Exerc..

[30] Joyner M.J., Coyle E.F. (2008). Endurance Exercise Performance: The Physiology of Champions. J. Physiol..

[31] Taylor S.J., Whincup P.H., Hindmarsh P.C., Lampe F., Odoki K., Cook D.G. (2001). Performance of a New Pubertal Self-Assessment Questionnaire: A Preliminary Study. Paediatr. Perinat. Epidemiol..

[32] McKay A.K.A., Stellingwerff T., Smith E.S., Martin D.T., Mujika I., Goosey-Tolfrey V.L., Sheppard J., Burke L.M. (2022). Defining Training and Performance Caliber: A Participant Classification Framework. Int. J. Sports Physiol. Perform..

[33] Williams N. (2017). The Borg Rating of Perceived Exertion (RPE) Scale. Occup. Med..

[34] Tanaka H., Monahan K.D., Seals D.R. (2001). Age-Predicted Maximal Heart Rate Revisited. J. Am. Coll. Cardiol..

[35] Castañeda-Babarro A. (2021). The Wingate Anaerobic Test, a Narrative Review of the Protocol Variables That Affect the Results Obtained. Appl. Sci..

[36] Jaafar H., Rouis M., Coudrat L., Attiogbé E., Vandewalle H., Driss T. (2014). Effects of Load on Wingate Test Performances and Reliability. J. Strength Cond. Res..

[37] Hebestreit H., Meyer F., Htay-Htay, Heigenhauser G.J.F., Bar-Or O. (1996). Plasma Metabolites, Volume and Electrolytes Following 30-s High-Intensity Exercise in Boys and Men. Eur. J. Appl. Physiol. Occup. Physiol..

[38] Tanaka H., Shindo M. (1985). Running Velocity at Blood Lactate Threshold of Boys Aged 6-15 Years Compared with Untrained and Trained Young Males. Int. J. Sports Med..

[39] Paterson D.H., Cunningham D.A., Bumstead L.A. (1986). Recovery O_2_ and Blood Lactic Acid: Longitudinal Analysis in Boys Aged 11 to 15 Years. Eur. J. Appl. Physiol. Occup. Physiol..

[40] Fellmann N., Bedu M., Spielvogel H., Falgairette G., Van Praagh E., Jarrige J.F., Coudert J. (1988). Anaerobic Metabolism during Pubertal Development at High Altitude. J. Appl. Physiol..

[41] Mero A. (1988). Blood Lactate Production and Recovery from Anaerobic Exercise in Trained and Untrained Boys. Eur. J. Appl. Physiol. Occup. Physiol..

[42] Wilson J.M., Loenneke J.P., Jo E., Wilson G.J., Zourdos M.C., Kim J.-S. (2012). The Effects of Endurance, Strength, and Power Training on Muscle Fiber Type Shifting. J. Strength Cond. Res..

[43] Tesch P.A., Thorsson A., Kaiser P. (1984). Muscle Capillary Supply and Fiber Type Characteristics in Weight and Power Lifters. J. Appl. Physiol..

[44] Foster C., Costill D.L., Daniels J.T., Fink W.J. (1978). Skeletal Muscle Enzyme Activity, Fiber Composition and VO_2_ Max in Relation to Distance Running Performance. Eur. J. Appl. Physiol. Occup. Physiol..

[45] Costill D.L., Daniels J., Evans W., Fink W., Krahenbuhl G., Saltin B. (1976). Skeletal Muscle Enzymes and Fiber Composition in Male and Female Track Athletes. J. Appl. Physiol..

[46] Bergh U., Thorstensson A., Sjödin B., Hulten B., Piehl K., Karlsson J. (1978). Maximal Oxygen Uptake and Muscle Fiber Types in Trained and Untrained Humans. Med. Sci. Sports.

[47] Chatel B., Bret C., Edouard P., Oullion R., Freund H., Messonnier L.A. (2016). Lactate Recovery Kinetics in Response to High-Intensity Exercises. Eur. J. Appl. Physiol..

[48] Mota M.R., Dantas R.A.E., Oliveira-Silva I., Sales M.M., da Costa Sotero R., Espíndola Mota Venâncio P., Teixeira Júnior J., Chaves S.N., de Lima F.D. (2017). Effect of Self-Paced Active Recovery and Passive Recovery on Blood Lactate Removal Following a 200 m Freestyle Swimming Trial. Open Access J. Sports Med..

[49] Sharma L., Hussain M., Verma S. (2017). Effect of Recovery Modalities on Blood Lactate Clearance. Saudi J. Sports Med..

